# Task-induced pupil response and visual perception in adults

**DOI:** 10.1371/journal.pone.0209556

**Published:** 2018-12-26

**Authors:** Antoinette Sabatino DiCriscio, Yirui Hu, Vanessa Troiani

**Affiliations:** 1 Geisinger Autism & Developmental Medicine Institute, Lewisburg, PA, United States of America; 2 Geisinger Center for Health Research, Danville, PA, United States of America; Brigham Young University, UNITED STATES

## Abstract

We assessed whether there are differences in pupil response that underlie the selection of local vs. global parts of a stimulus array in healthy adults. We designed a Navon Figures eyetracking paradigm (i.e. large figure composed of small figures), requiring an individual to vary only the information attended to within an image. We found that participants have a characteristic constriction of the pupil waveform during selection of local information relative to global information. Because stimuli and lighting conditions were identical across conditions, this indicates that pupil changes may serve in a visual filtering mechanism important for attentional selection. This work represents the first characterization of pupil response in the context of selective attention, suggesting that mechanisms underlying the earliest stages of visual processes could be relevant for perception and visual selection.

## Background

An individual’s visual environment consists of a vast array of individual, detailed elements (i.e. local information) arranged within a broader, contextual configuration (i.e. global information). Within an experimental setting, paradigms using hierarchical stimuli composed of smaller forms within larger forms can be used to objectively assess the perceptual extraction of local features relative to their global whole [1]. An individual is required to attend to either the larger, global figure, or the smaller, local form, as quickly as possible. Within behavioral versions of this type of task, a global precedence effect has been found [1,2]. Neurophysiological and neuroimaging studies have revealed important information regarding cortical regions and structural changes associated with global visual processing [3–5]. While methods such as these have been successful for understanding the potential neurobiological mechanisms subserving visual perception and global-local processing, psychophysiological methods such as eyetracking and pupillometry offer a non-invasive and inexpensive peripheral index of underlying neural function.

Pupillometry (the measurement of pupil size) has an enduring (~50 years) history of using pupil response as a peripheral index for cortical feedback and subcortical function in psychological and neurobiological research [6–8]. It has long been established that changes in pupil diameter reflect a basic physiologic response of the pupil due to changes in ambient light, color, spatial frequency, and movement. Simple changes in lighting conditions are known to activate the pupillary light reflex (PLR), which serves to automatically adapt to changes in light levels. Beyond the basic PLR, research has highlighted the influence of various factors (i.e. arousal, cognitive effort or cognitive load, decision making, interest, emotion) on observable changes in pupil size [9–11]. This research has demonstrated that the pupil does not solely represent a basic, low-level visual reflex to light but also a complex physiologic response that is also known to be influenced by visual attention and the awareness, interpretation, and allocation of attention to any given target.

There have been several studies that link pupil responses with changes in visual attention. For example, covertly attending to a bright stimulus as compared to a dark stimulus elicits pupil constriction [12]. Similarly, pupil size is modulated when covertly attending to a dark or light stimulus and size of the pupil change predicts behavioral measures of task performance in a classic attentional cueing paradigm [13]. Other work has shown that the amplitude of pupil oscillations between dilation and constriction follow visual attention allocation [14]. Daniels et al. [15] reported an association between changes in attentional spread and pupil metrics, with alternating states of more broad and focused attention coinciding with larger and smaller pupil diameter, respectively. Thus, these studies suggest that pupil diameter can affect visual input at early stages of visual processing and potentially optimize perceptual or attentional strategies [12–15] or otherwise create spherical aberrations that can adjust attentional focus [15]. Overall, this pupil-specific work also aligns with behavioral research that demonstrates that attention can modulate visual salience, selection, and perception [16,17].

There is currently no research characterizing changes in task-induced pupil response within the context of global-local processing and shifts in attentional focus. In the current study, we assess whether the visual selection of global and local features of a hierarchical stimulus require changes in attentional focus that are reflected in differences in pupil response. Given the current knowledge of global-local processing biases, we wanted to [1] investigate global-local perception in healthy adults and [2] explore differences in patterns of dilation and constriction within task-induced pupil response that may subserve observable differences in broad, global visual perception versus narrowed, local visual perception.

We assessed pupillometry while healthy adults performed a task using Navon figures. Based upon previous work demonstrating changes in pupil constriction and dilation that accompany the broadening or narrowing of attention focus [15], we hypothesized that local information processing would require increased focus and global processing a more relaxed focus, which would be reflected in dynamic changes in pupil response curves over the course of a trial period. We also explored potential differences in baseline pupil diameter prior to stimulus onset, which may further indicate a preparatory attention effect. Recent work has shown that the anticipation of a particular stimulus property (i.e. brightness) induces pupil constriction [18,19],other work that has demonstrated pupil modulations based on anticipated and perceived changes in low- and high-level stimulus content [13,18–20], and a sustained pattern of pupil dilation that was interpreted to reflect the content of the upcoming decision [21].

## Materials and methods

### Task and stimuli

**Fig 1:**
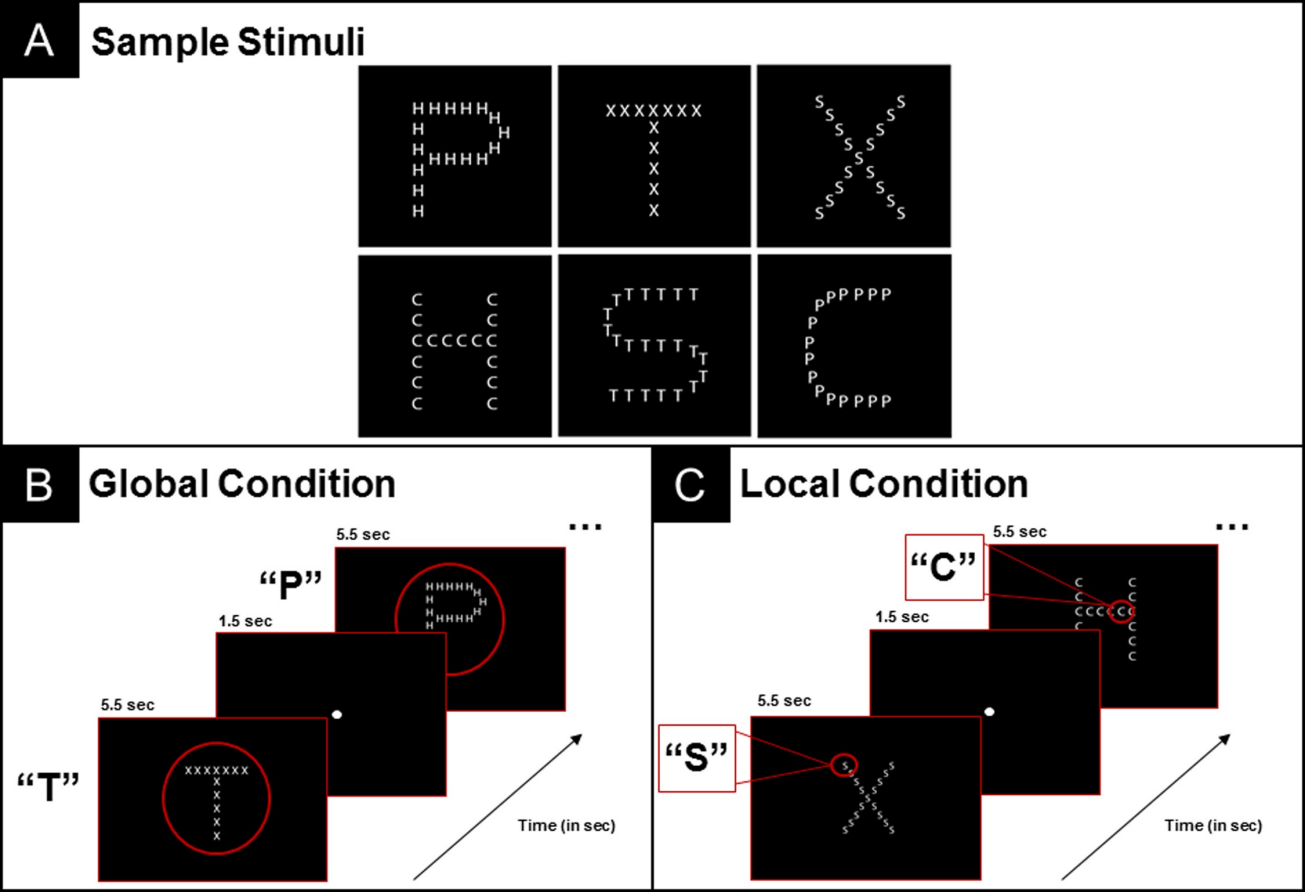
Sample Navon figure stimuli. (A): Each figure was hierarchically composed of a larger letter made up of smaller letters (letters included were 'X', 'P', 'T', 'S', 'H', and 'C'). (B): Two sample trials (global condition) separated by an interstimulus fixation period (1.5 sec). (C): Two sample trials (local condition) separated by an interstimulus fixation period (1.5 sec).

**Fig 2:**
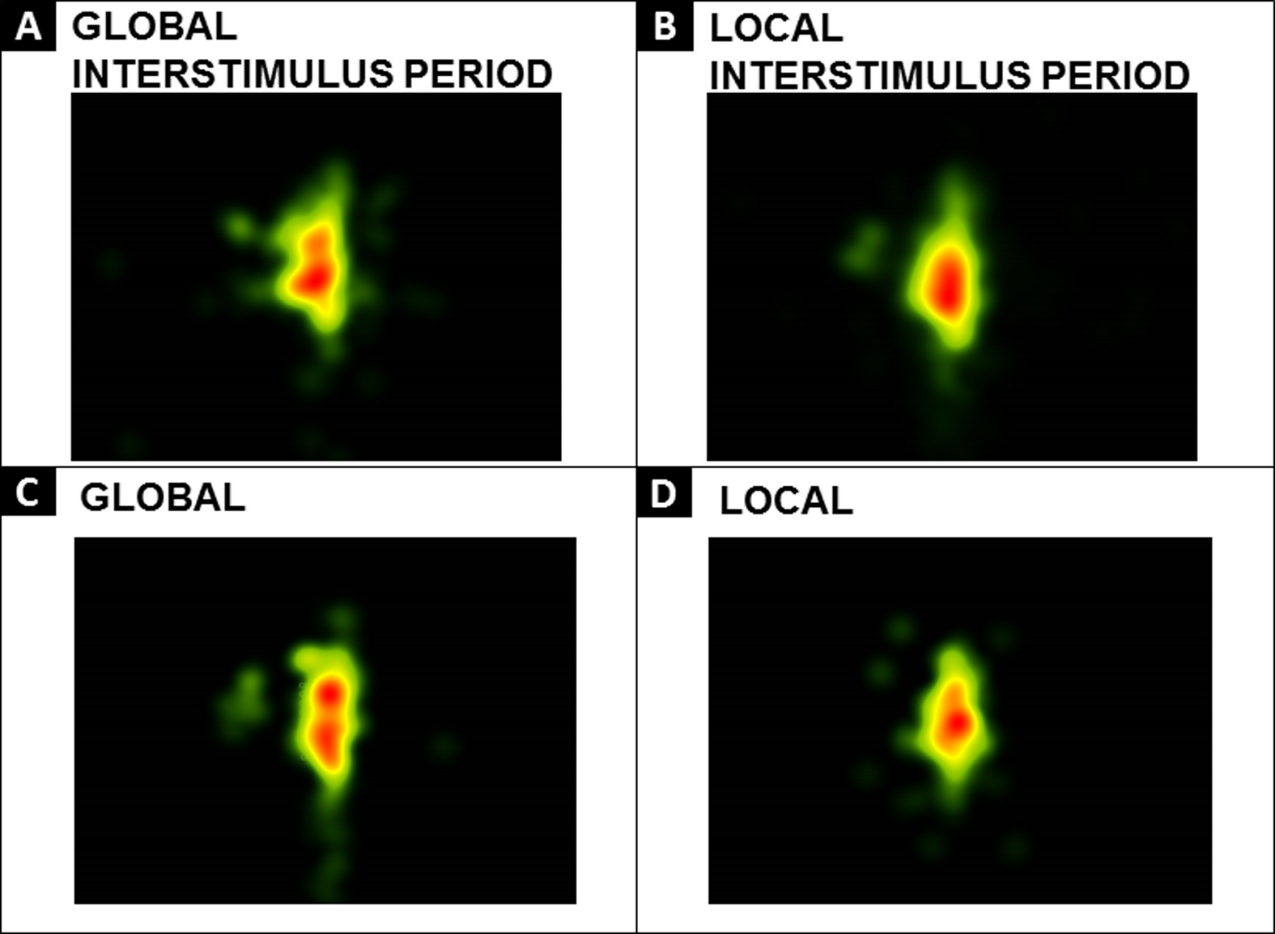
Heat maps. Group-level heat maps illustrated above averaged across the interstimulus fixation period as well as the trial period confirm that participant’s gaze remained centralized. (Panel A: Global Interstimulus Period; Panel B: Local Interstimulus Period; Panel C: Global Condition; Panel D: Local Condition).

Twenty-one Navon Figures [1,2] were created. Each figure was hierarchically composed of a larger letter made up of smaller letters. Letters included were 'X', 'P', 'T', 'S', 'H', and 'C'. Sample stimuli can be seen in Fig 1; Panel A. All stimuli were 2x2 inches in total size and subtended no larger than a 2.5° visual angle from center of the screen. Stimuli were presented on a black background and elements within each hierarchical stimulus were colored gray to minimize the amount of luminance emitted from the screen (i.e. a white background would have more luminance). The stimuli used in the global and local condition were identical and thus luminance was equal across both conditions. While the luminance properties of our stimuli are defined by incremental changes in luminance against a black background (i.e. the appearance of Navon figures on a black screen), the same stimuli and experimental parameters were implemented across both global and local conditions. Thus, the same incremental changes in luminance in one experimental block for one condition would be identical to those that take place over the course of another. This served as a control for low-level visual features such as luminance that impact pupil changes. Participants varied only the information attended to within each figure based upon task instructions and visual stimulus properties did not differ between global and local conditions.

**Fig 3:**
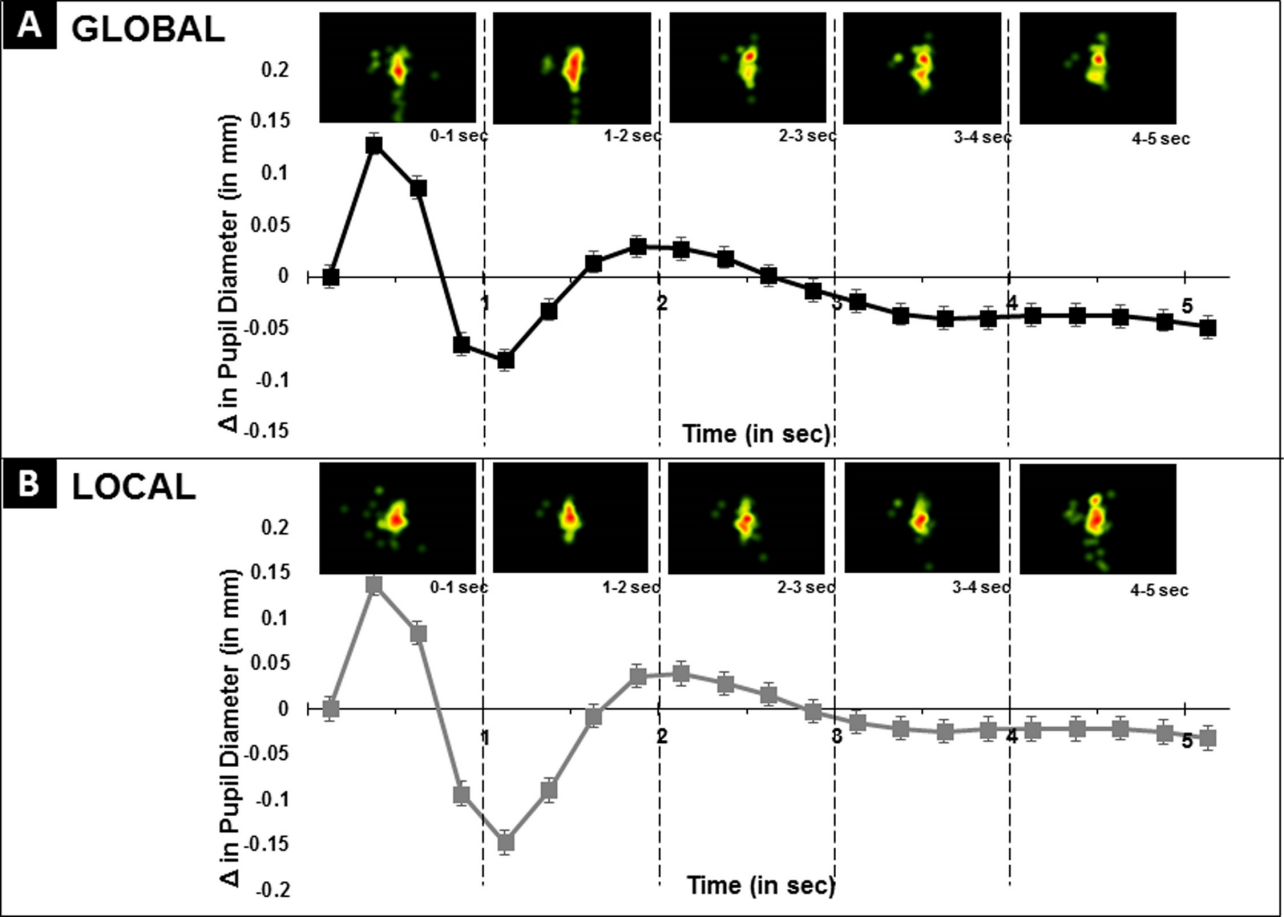
Heat maps. Group level heat maps extracted at 1-second intervals for an example trial for both conditions (Panel A: Global; Panel B: Local).

Participants were required to identify either the larger (global) letter or the smaller (local) letter, in separate blocks. Thus, each participant completed one block of one condition and then completed a second block of the alternate condition (i.e. conditions were *not* mixed within each block). Completion order of global and local blocks was counterbalanced across participants; however, trial order was not randomized within blocks. To obtain the most accurate pupil metrics and to minimize visual saccades that have previously been found to influence pupil metrics [22–24], participants were discouraged from looking around the screen and were instructed to keep eyes focused in the central region where stimuli were displayed. Group-level heat maps were generated in Tobii Studio in order to confirm that participant’s gaze remained centralized. Heat maps summarize fixations in regions across the stimulus as well as indicate the length of fixations in that region (red indicating the highest number of fixations for the longest time). Group-level heat maps from across the interstimulus fixation period as well as the trial period (see Fig 2) indicate visual saccades were indeed minimized and participants maintained focus in the central portion of the screen where stimuli were presented. Fig 3 includes group-level heat maps extracted at 1-second intervals from an example trial for both global and local conditions (generation of pupil response waveforms described below). These 1-second interval heat maps further illustrate that participants maintained focus throughout the stimulus presentation. See Figs 2 and 3.

Participants were given five sample items, administered via PowerPoint on a separate laptop computer or a flipbook composed of sample stimuli, to ensure they understood task instructions and reached proficiency before beginning each block. These sample items were administered under normal testing conditions (i.e. in the same dimly lit room) prior to calibration (see description of calibration procedure below). Sample items included an interstimulus fixation (black screen with a centered small white dot) to prompt participants to keep eyes focused in the central region of the screen. This interstimulus fixation was removed from experimental blocks and only a blank screen (black screen that matched the black background of each stimulus) was used in order to (1) allow pupil diameter to return to baseline and (2) avoid a priming effect of having a stimulus present (i.e. a circle or small crosshair “+”) before the presentation of each hierarchical stimulus. In other words, we did not want the presence of a similar shape or letter stimulus during the interstimulus period to confound behavioral and task-induced pupil response within each trial. Despite the lack of an interstimulus fixation, participants were largely able to maintain focus on the central region of the screen in between each trial. Each experimental block consisted of 21 trials. Participants were given a short break in between each block and reminded of the instructions for the upcoming block. Previous reports of eye tracking methods have reported more pronounced visual saccades and variable eye gaze patterns in the early stages of an experiment as a participant is adjusting to task demands, stimuli, and experimental setup [25]. Thus, the first trial of each block was eliminated from analysis due to aberrant pupil response and visual saccades noted across participants; thus leaving 20 trials for each condition.

Each trial consisted of two events: a interstimulus period (1500 ms), followed by a 5500 ms stimulus presentation period (see Fig 1, Panels C and B). Participants received the following instructions, “You are going to see letters on the screen. These letters are going to look like big letters made up of tiny letters.” For the global block, participants were told “You will need to ignore the tiny letters and only identify the larger letters”. For the local block, participants were told “…ignore the larger letters and only identify the smaller letters.”

### Participants

Forty-three individuals participated in the study. All participants reported never receiving a clinical diagnosis of a neurodevelopmental disorder, psychosis, mood disorder, or documented visual spatial disorder/impairment or delayed visual maturation. All participants had normal and/or corrected-to-normal vision. Participants confirmed that they had no history of previous drug or alcohol. Participants completed the Wechsler Abbreviated Scale of Intelligence-2^nd^ Edition (WASI-II) [26] on the same day of testing. Adults were primarily recruited from the surrounding community, a local university, and a major health system where the research was taking place. All research practices were approved by Geisinger’s internal ethics review board. Written consent was obtained from all research participants.

Data from a total of N = 33 participants (of the original 43 recruited and enrolled) were included in the final analysis (mean age in years = 21.18 +/- 3.24; 17 males). Demographic profiles as well as average full scale IQ (FSIQ) for each group are presented in Table 1. Participants with >40% of missing data across *both* conditions (equivalent of approximately 11 or more total trials with a significant proportion of missing data) were excluded from analysis. Thus, for data to be included in analyses, a minimum of 40% of global trials and 40% of local trials needed to be obtained. This criteria resulted in the exclusion of ten participants from analyses due to fixation errors during eyetracking recording, leaving the final sample of N = 33 described above.

**Table 1:** Means (SDs) of demographic and symptom data.

	N = 33
**Age (years)**	21.18 (3.24)
**Age range (years)**	18–29
**Number male (% of sample)**	17 (51.5)
**FSIQ**	112.9 (10.45)

### General procedure and apparatus

Eye movements and pupil diameter were recorded using a Tobii X120 binocular eyet racking system (Tobii Technology AB, Danderyd, Sweden), which records eye gaze as well as pupil dilation. The system is a stand-alone eyetracking unit that monitors eye gaze patterns and pupil diameter at rate of 60 Hz by using infrared light to produce reflection patterns on the corneas. The eyetracker monitors the movements of these reflections relative to eye position. Multiple sensors assess eye movements and pupil diameter using bright and dark tracking. Tobii eye trackers adjust pupil measurements based upon measured distance between the eye and the sensor in order to accurately measure pupil size. Individual measurements regarding the position of the eyes and optical distortions between the cornea and the lens and other gaze artifacts (i.e. blinks and head movement) are accounted for as a part of the Tobii recording. Stimuli were presented on a 21.5-inch display monitor via EPrime using the Tobii extensions that allowed for concurrent eye gaze monitoring and pupillometry data acquisition.

Testing was done in a quiet, darkened room separate from an experimenter control room via a wall with a two-way mirror. All participants were positioned at a distance of 55–65 cm from the display screen and completed the standard Tobii Studio, 5-point calibration procedure prior to the start of testing. During our calibration procedure, we ensured that each person was within this range as well as monitored each participant’s distance from the screen throughout eye tracking recording. A Symetrix Solus 4 audio mixer allowed for communication between the participant and experimenter(s) between the two rooms. Gaze behavior and eye position was monitored throughout the testing session on a separate monitor in the experimental control room to ensure continuous data collection.

### Eyetracking data and analysis

**Fig 4:**
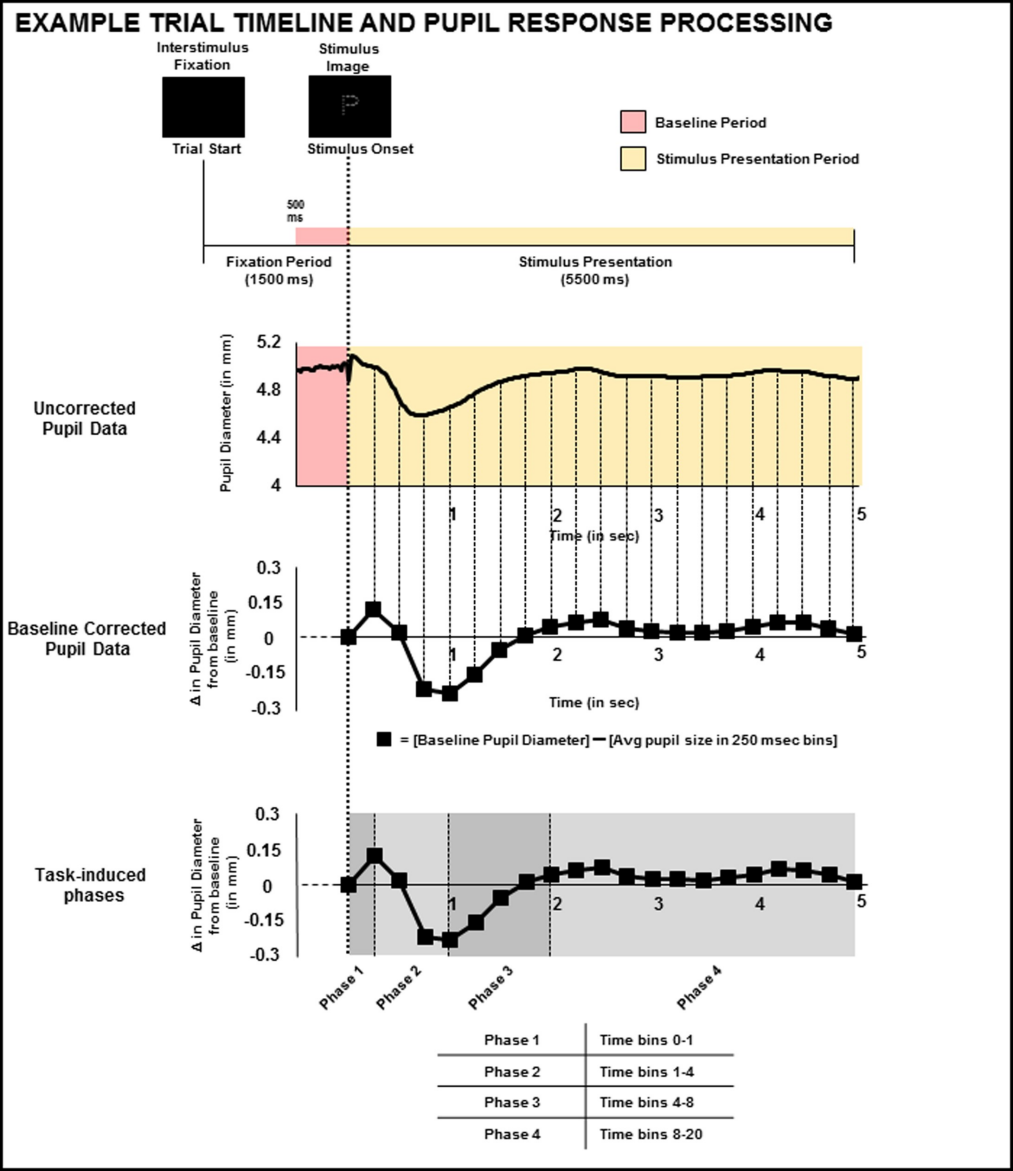
Pupil response data processing. **(**First from the top) A timeline depiction of a single trial with interstimulus fixation period and stimulus presentation with indication of 500 msec immediately (red) prior to stimulus onset and presentation (yellow) from which average baseline pupil diameter was extracted for each trial. (Second) Uncorrected pupil response waveform, following interpolation and filtering, representing one trial from one participant. Average baseline pupil diameter for each trial was extracted from 500 msec immediately preceding stimulus onset. (Third) Average pupil size was computed every 250 ms and plotted over time to obtain an average pupil waveform for each condition (global and local). Change in pupil diameter from average baseline diameter, was computed (difference between each value obtained within every 250 ms bin and baseline pupil diameter). (Fourth) Time bins within the pupil response waveform were divided into four phases. All relevant pupil data has been made available in a .csv file in the supplement.

Data was exported from Tobii Studio and subsequent processing and analysis proceeded using adapted MATLAB scripts [27], R [28,29], and SPSS. In the event of missing data from one pupil, missing values were replaced with the recorded value for the other eye. To deal with missing samples from both eyes (blinks, tracking errors), pupil response for each block was smoothed using a low pass (15 Hz) filter and then a linear interpolation was used to fill in gaps. Values from both eyes were then averaged. Baseline pupil diameter was extracted using the average from 500ms immediately preceding stimulus onset for each trial (see Fig 4). Pupil diameter for each trial was calculated by first segmenting the 5s trial window into 20, 250ms time bins. There was consistent, minor variability in Tobii eye tracking acquisitions across all participants (some having slightly over and/or under 330 samples taken across each of the 5.5 second stimulus presentations). Thus, the initial 300 (5 seconds) collected samples within each trial were maintained in order to establish consistent and continuous data across participants and were used in analyses described below.

Raw pupil diameter was averaged for each 250ms time bin. We then produced baseline corrected, task-induced pupil waveforms by subtracting the average pupil diameter extracted from the period immediately preceding stimulus onset. The average pupil diameter used was condition-specific (i.e. for global trials, the average baseline pupil diameter was determined from the 500 ms period immediately preceding global trials and vice-versa for local trials). These condition-specific pupil averages were then subtracted from each 250ms time bin (see Fig 4) similar to previous eye tracking research on task-induced pupil response [30]. This process served to produce baseline corrected, task-induced pupil waveforms that reflect changes in pupil diameter following stimulus onset [31–38]. Baseline corrected, pupil waveforms were divided into four phases based upon previous research that has described a phase based response in pupil changes (See Fig 4) [30]. Phase 1 (time bins 0–1) was an obvious dilation of the pupil immediately following stimulus onset. Phase 2 (time bins 1–4) was a rapid constriction of the pupil. Phase 3 (time bins 4–8) was a slow increase in pupil diameter towards baseline. Finally, Phase 4 (time bins 8–20) extended to the end of the trial period. In order to objectively define the change in pupil response and illustrate sensory driven, functional changes in pupil dilation and constriction within each of these phases, we calculated each phase based upon the first and last time bin over comparisons of discrete values that would result from simple averages.

In addition to pupil metrics, primary dependent measures included accuracy and reaction time (RT) (via responses that were spoken aloud). As mentioned above, participants were instructed to clearly state the answer (i.e. either the global or local element within each stimulus depending on the experimental block). Accuracy was determined based on whether the correct element was identified and provided via verbal response based on task instructions. A Serial Response (SR) box and Audacity (audacityteam.org/about/) were used to record utterances during each testing session to ensure accurate RT was obtained. The SR box records the time at which a vocal response is made for each trial; however, any audible noise registered by the SR box is recorded as a response (which includes participant sighs, coughs, and non-response “ums”, for example). Thus, Audacity was used as the primary source for recording and obtaining time of response. RT was determined by subtracting the onset time of the correct utterance from the flagged stimulus onset. Stimulus onset was signaled via a 500 ms, 100 Hz tone at the start of each trial, audible only to the experimenter(s) in the control room during testing. Responses recorded via the SR box were used for confirming RTs recorded via Audacity. This procedure allowed participants to respond in a naturalistic way as soon as soon as the correct answer was identified. We chose this procedure (rather than a potentially more traditional button response) in order to prevent additional cognitive effort or stimulus-response mapping that would be necessary to remember the various letters used in our stimuli.

## Results

### Behavioral results

We first analyzed accuracy and RT results, in order to assess whether the stimulus versions were matched on difficulty and to investigate the presence of a global/local bias. Our behavioral dependent measures deviated from a normal distribution according to a Shapiro-Wilk test for normality. Accuracy for global and local conditions demonstrated non normal distributions (*p*’s<0.0001). RT for global and local conditions also deviated from a normal distriution (*p*’s<0.009). Results from nonparametric, *sign tests* were used to assess within group differences for accuracy and RT in global and local conditions. There was no significant within group differences in task accuracy between conditions according to nonparametric, exact sign tests (mean accuracy for global condition = 99.8 ± 0.01, min: 95.2, max: 100; mean accuracy for local condition = 99.8 ± 0.01, min: 95.2, max: 100, *p*>0.95, NS). Thus, participants were equally accurate across both conditions.

In order to test our a priori hypotheses regarding perceptual biases and previous reports of global precedence (ease of extracting global information as compared to local), a nonparametric, exact sign test was used to compare the differences in RT in global and local conditions. RT when identifying global information (mean RT for global = 0.81 ± 0.16, min: 0.62, max: 1.33) was significantly faster as compared to identifying local information (mean RT for local = 1.20 ± 0.68, min: 0.67, max: 3.09) (*p*< 0.0001). This indicates that participants showed a global bias.

### Baseline pupil diameter

As mentioned previously, baseline diameter was determined based upon the 500 ms interval immediately preceding stimulus onset for each trial. Previous work has used various intervals to determine baseline pupil diameter (including both shorter intervals (200 ms) and longer intervals (1000 ms)) [31–38]. Within the current experimental design, there is risk that residual visual stimulation could alter baseline pupil diameter especially in the time immediately following stimulus offset. Thus, we chose to extract baseline towards the end of the interstimulus fixation period and in the final 500 ms before the next trial began. Previous studies have highlighted a variety of factors that influence baseline as well as task-based pupil diameter (i.e. ambient light, stimulus properties, arousal sate). Research has indicated variability in baseline pupil diameter associated with experimental conditions [39]. Because our task-related pupil waveforms are baseline-corrected using condition-specific baseline values, we wanted to ensure that baseline differences weren’t artificially causing us to interpret condition or group differences in the baseline corrected pupil waveforms. By doing this, we insure that there are not differences in task-related changes that are an artifact of differences in baseline between the two conditions. That is, if baseline was consistently larger in the local condition and smaller in the global condition but there were no differences in raw (pre baseline-corrected) task-induced pupil response observed, a difference in baseline between the conditions could lead to erroneous conclusions based on condition-specific baseline correction. Thus, before moving on to task-related changes in pupil response, we compared measures of baseline pupil diameters extracted from rest periods prior to trial onset for each trial and averaged for each condition.

**Fig 5:**
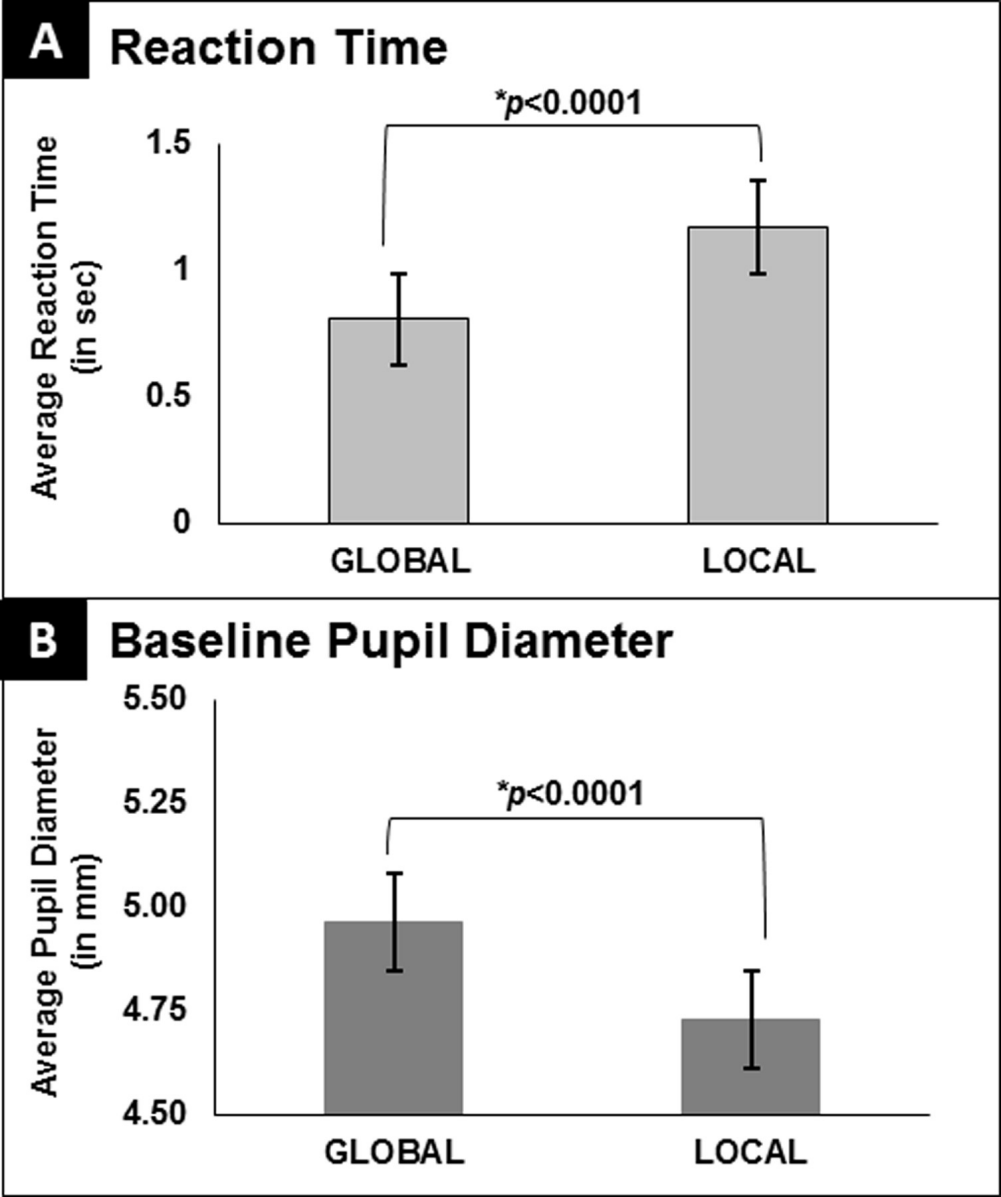
Behavioral results and baseline pupil diameter. (A) Group differences in average reaction time (in sec) for the global and local conditions. (B) Group differences in average baseline pupil size (in mm) for the global and local conditions. Error bars indicate standard error of the mean.

Baseline measures for global and local conditions were normally distributed according to a Shapiro-Wilk test of normality (*p*’s>0.20, NS) and no outliers were identified. See Fig 5, Panel B. Baseline pupil diameter was significantly larger for global relative to local blocks (*t*(32) = 4.48, *p*<0.0001). Thus, participants demonstrated a relatively smaller baseline pupil diameter overall as well as a significant difference in global relative to local baseline pupil diameter. These results highlight a preparatory attention effect as participants demonstrated a larger baseline pupil diameter in global condition and a smaller baseline pupil diameter for the local condition.

### Task-induced changes in pupil size

**Fig 6:**
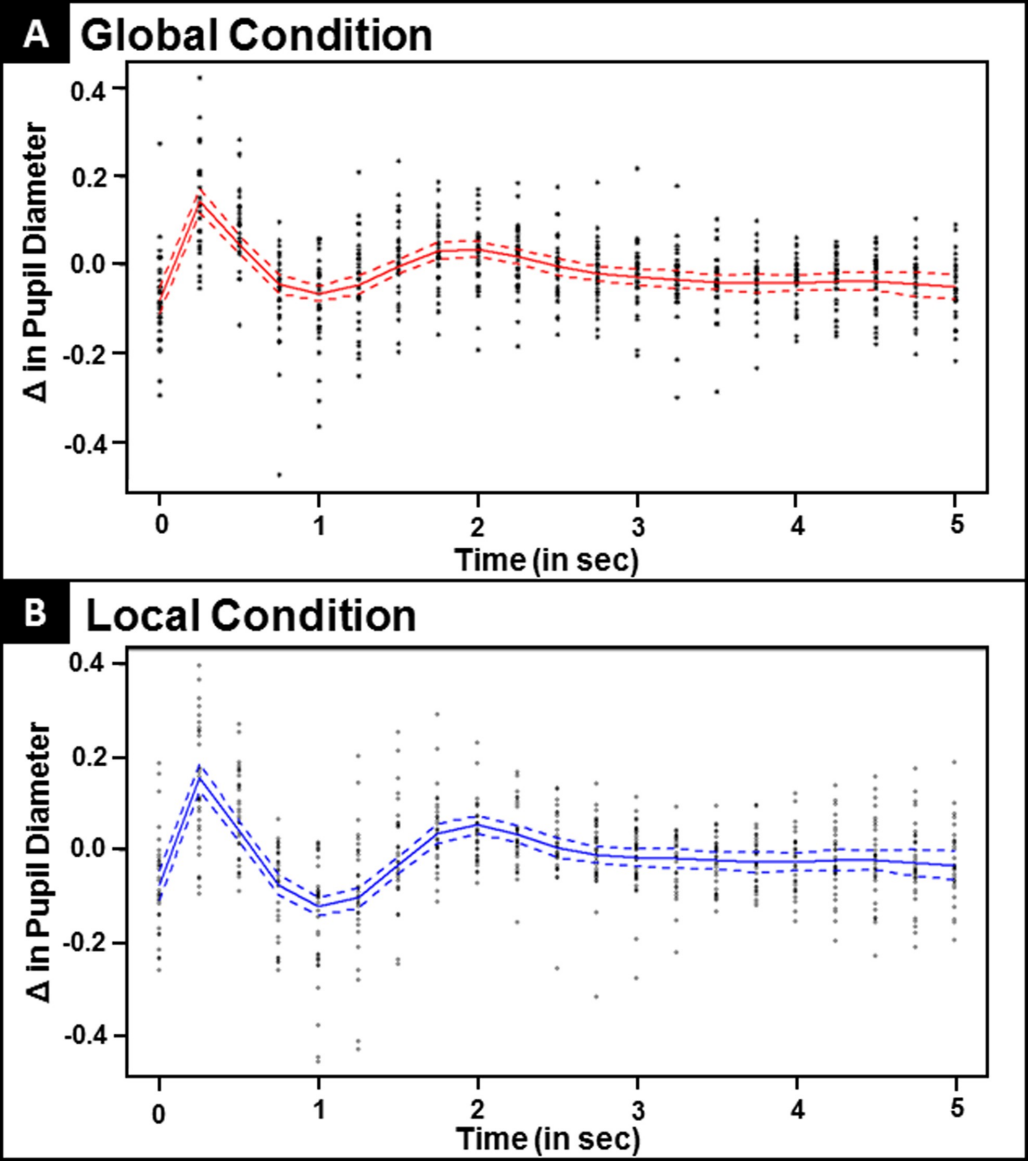
(A) Pupil waveforms depicting task-induced pupil response during the global condition. (B) Pupil waveforms depicting task-induced pupil response during the local condition.

We next examined differences in the task-induced pupil waveforms and compared these pupil response waveforms between global and local conditions. As described previously, pupil waveforms were computed across 20, 250 ms time bins over the course of the 5 s trial period. A cubic spline was fit for both global and local conditions (see Fig 6, Panels A and B). Pupil response waveforms were then divided into 4 task-induced phases based upon descriptions of task-induced changes in pupil response in previous research [30]: Phase 1 (time bins 0–1), Phase 2 (time bin 1–4), Phase 3 (time bin 4–8), Phase 4 (time bin 8–20) (see Fig 2 for depiction of phases). Changes within each phase were then quantified as a singular value based upon the difference in pupil diameter from the start and end each phase (i.e. for Phase 1, the difference between time bin 0 and 1, for Phase 2, the difference between time bin 1 and 4, for Phase 3, the difference between 4 and 8, and so on). The difference in pupil diameter changes between global and local conditions within each phase were normally distributed according to Shapiro-Wilk test of normality (*p*’s>0.18). Paired t-tests with bootstrap tests were conducted in order to investigate differences in pupil response between global and local conditions for each of the four phases. Bootstrap p-values using 1000 resamples from original samples are presented to test robustness of results.

**Fig 7:**
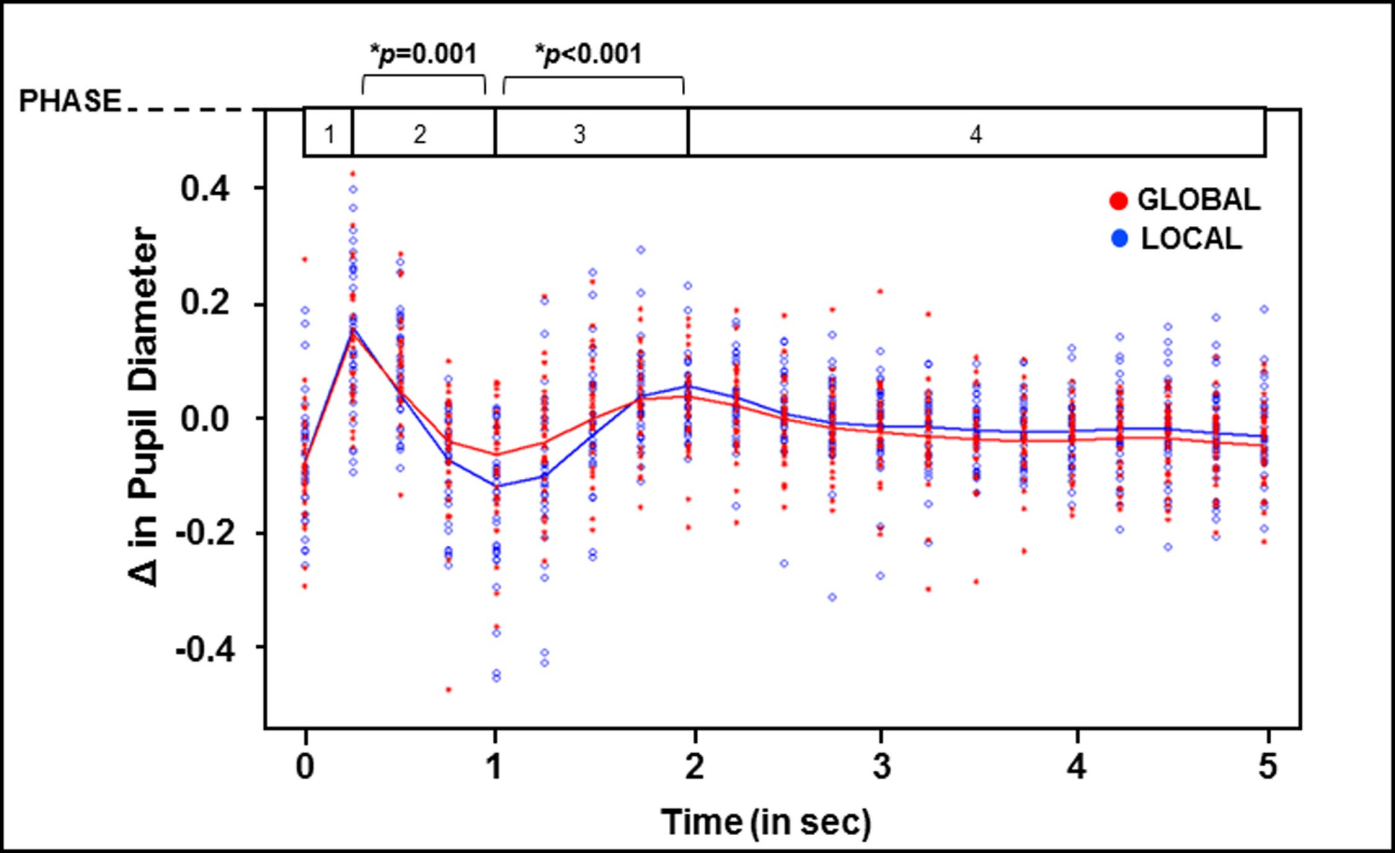
Task-induced pupil response. Pupil waveforms depicting task-induced pupil response during the global and local conditions. Phases are depicted at the top of the graph and significance is indicated.

See Fig 7 for results described here. In Phase 1 (time bin 0–1), change in pupil dilation in the initial stage of the pupil response waveform did not differ between global and local conditions (Global Δ Value: 0.21 mm, Local Δ Value: 0.21 mm; t(32) = -0.20, *p* = 0.81, NS). In Phase 2 (time bin 1–4), a rapid pupil constriction was observed in both conditions. This constriction was significantly larger for the local condition relative to the global condition (Global Δ Value: -0.21 mm, Local Δ Value: -0.29 mm; t(32) = 3.2, *p =* 0.001). In Phase 3, pupil response dilated across both global and local conditions. Once again, significant differences in Phase 3 of the pupil response were present between global and local conditions (Global Δ Value: 0.11 mm, Local Δ Value: 0.19 mm; t(32) = -5.0, *p*<0.001), with a larger change in pupil diameter in the local condition relative to the global condition during this phase. In the final segment of the pupil response waveform (Phase 4), change in pupil size did not differ between conditions (Global Δ Value: -0.08 mm, Local Δ Value: -0.07 mm; t(32) = -0.23, *p* = 0.82, NS). These results indicate that, when selecting local relative to global information, participants displayed a significant difference between conditions and demonstrated greater constriction during Phase 2 followed by greater dilation during Phase 3 for local trials relative to global trials.

Given the fast initial dilation in pupil response during Phase I (time bin 0–1), we also explored whether eye blinks were interrupting our pupil measurements and causing an artifact in our reported pupil response data during this time bin. In pupillometry research, eye blinks are typically handled as segments of missing data since the pupil is occluded. Various interpolation or estimation methods are then implemented to fill in these segments and approximate pupil response during periods of data loss. Thus, we wanted to determine whether eye blinks during the baseline period were resulting in data loss that would cause an artifactual pupil dilation during the first time bin. Blinks were defined as continuous periods of missing eye gaze data of at least 100 msec and >500 msec. This definition of eye blinks was based upon previous eye tracking research and established infrared eye tracking norms [40].

We extracted eye blinks from the baseline period and counted the number of eye blinks registered within this period for all trials. We found that no eye blinks occurred during the baseline period for >70% of the trials, indicating that only a small degree of data loss occurred during the baseline period. In addition to determining the overall data loss due to blinks occurring during the baseline period across all trials for all subjects, we also identified subjects that were outliers based on number of blinks. Outlier subjects were defined as individuals with an eye blink count ≥ 2 standard deviations from the group average. We repeated our analysis with those individuals (n = 3) excluded. Results were identical to those reported above and have been included as a supplement. Thus, while eye blinks result in segments of missing data that can impede pupil measurements, these findings confirm that eye blinks in the current study did not result in an artifact in reported pupil response data.

## Discussion

The current study aimed to better understand the processes that underlie differences in visuospatial selection. We find differences in pupil response when identifying local information compared to global information. This form of pupil response waveform has not previously been described in the context of a global and local processing task.

Participants demonstrated a global bias, in that they were faster to report global information, consistent with previous reports of a global bias in adults [1,2]. In addition to faster RTs for the global condition, average baseline pupil diameter (measured immediately before each trial) was larger (more dilated) prior to global trials than local trials. Differences between global and local selection in components of the pupil waveform indicate a noticeable constriction within the pupil waveform when selecting local information relative to global information. Thus, both baseline metrics and waveform data in adults consistently indicate that the pupil is more constricted for local versus global processing, both prior to trial onset and during the active selection process. This point is important, as there is a great deal of pupillometry work that associates pupil dilation with increased effort. From an effort-based framework, using the reaction time data alone, one might have expected that because global processing is relatively ‘easier’ based on faster reaction times in the adult population, that baseline pupil and dynamic task-induced pupil changes would be more dilated for local processing, because the local condition is more ‘difficult’. We find the opposite, with a significant difference in pupil changes as a result of pupil constriction for local relative to global. Other research has highlighted the continuous influence of modulatory systems controlling pupil dilation throughout decision making tasks. Gee et al. [21] reported a sustained pattern of pupil dilation that was interpreted to reflect the content of the upcoming decision as well as individual bias. In the current study, our finding of larger baseline pupil diameter for our global condition may reflect a sustained, attentional state during the block of global condition trials that reflects the upcoming decision (identification of the global form) and is related to response bias. The current result is more in line with the growing body of evidence that finds pupil changes associated with covert attentional processes to mimic those of a reflexive, sensory response.

It has been shown that changes in pupil diameter can also be brought under voluntary control via cognitive mechanisms, cortical feedback, and subcortical function involved in strategic search and active cognitive or attentional filtering. For example, when covertly attending to a bright vs. dark stimulus, the pupil constricts, even when the visual input is the same [9,12,13,41]. Daniels et al. [15] demonstrated, via an attentional cueing paradigm, that changes in pupil diameter influences spherical aberrations that can either [1] broaden or spread attentional focus and blur the retinal image as the pupil dilates or [2] narrow attentional focus and bring a retinal image into detailed view as the pupil constricts. This work indicates that the pupil can function as a visual filter prior to (or even in the absence of) physical changes in a stimulus. Because the stimuli and luminance were identical in our two conditions and stimuli were small enough to prevent eye movements, we interpret these results as the attentional modulation of the pupil response. Even prior to trial onset, there seems to be an attentional process occurring that results in larger pupil diameter for global trials and smaller pupil diameter to local trials. In addition, dynamic changes of pupil size during the trial show an even greater constriction during local trials relative to global trials. Thus, this finding demonstrates that selecting smaller, local, bits of information relative to larger, global elements of a stimulus are associated with greater pupil constriction. The function of pupil constriction may move beyond a basic sensory reflex towards a mechanism associated with task-specific components of flexible spatial selection. Findings of greater constriction when identifying local information is aligned with previous research documenting alternating patterns of pupil constriction and dilation in contexts requiring shifts in the narrowing and broadening of the attentional window [14,15].

We would like to acknowledge that there are several theoretical constructs through which the current results can be interpreted and/or future work can help to delineate the precise underlying mechanisms of visual selective attention. The visual attention system has multiple mechanisms for rapidly processing relevant information, including (1) perceptual biases that can help process information via multiple strategies within the hierarchy of visual processing [42], (2) spatial frequency processing pathways and biases [43–45]; and (3) a somewhat flexible attentional window that can be broadened or focused based on current state [46,47]. It will be important for future work to investigate which of these strategies or processes and underlying neurobiological mechanism underlie this phenomenon.

Current analytic approaches in pupillometry research utilize data reduction methods that aggregate temporally rich pupil data and compare averaged data between conditions or groups. Often this involves comparing a finite variable from a fixed timepoint such as dilation, average pupil diameter, or average change of pupil diameter relative to a baseline measure or the start of a trial period [30,39,48–50]. These traditional forms of analyses overlook meaningful and dynamic changes in pupil diameter in response to sensory input. Pupil diameter changes over time, as a function of stimulation, and can be conceptually linked to a peripheral physiological signal that is tightly linked to underlying neural function (much like electroencephalography). Additionally, variability in pupil response between individuals highlights the need to characterize meaningful individual differences in pupil response that may be missed with more traditional forms of analyses. Here, instead of extracting one average pupil metric for each trial, we model temporal response data across trials and establish a pupil response trajectory for each condition. Continued use of this type of modeling and analysis will allow for future research with similar paradigms to assess a variety of pupil response differences that are not available with traditional analysis. For example, future studies can compare each participant’s trajectory to an established trajectory and allow for outlier detection at the trial and subject level. Research utilizing pupillometry and temporally rich eye tracking data should focus analyses on quantifying pupil response trajectories, describing functionally relevant pupil response patterns, and characterizing variability in pupil response as a key indicator of perceptual strategy, cognitive ability, and underlying neural function.

Finally, we have documented and described an early dilation phase within our pupil waveforms. Previous work has documented a similar pattern in pupil waveforms and this dilation has been described as a perceptual response to the occurrence of a stimulus [30], an orienting response [51], as well as an anticipatory dilation related to the onset of a stimulus and preparation of making an appropriate verbal response [52]. We suggest that future research continue to describe temporal changes in task-induced pupil response in order to more accurately characterize patterns of dilation and constriction that may result from differing task demands or stimulus properties.

There are several limitations within the current study that should be addressed in future research. Our paradigm was administered in a block design with participants completing a series of trials in one condition before moving on to the next. Additional research is necessary to understand pupil changes in the context of switching from one condition to the other in a single experimental block. For instance, it remains unclear the degree to which the cognitive demand of preparing a response based upon a changing pre-stimulus cue might also influence task-induced changes in pupil diameter. Another limitation is that there were reaction time differences between the global and local conditions. Longer reaction times are generally associated with increased task difficulty and greater pupil dilation (i.e. effort-based framework). Changes in task-induced pupil response may also be due to the processes associated with planning and/or executing a behavioral response (i.e. verbal or manual) as compared to being a pure index of stimulus processing. For example, previous work has shown that increased pupil diameter is associated with identifying less frequent (i.e. more difficult) words. One study showed that this larger task-induced pupil diameter for low frequency words was present even with a delayed response version of the task, indicating that the effect was present even in the absence of reaction time differences [52]. Although the longer reaction times for local trials reported here cannot explain our novel finding of a significant pupil constriction within an effort-based framework, future research may need to account for the potential influence of preparing and delivering a response on the various phases of the pupil trajectories.

Finally, the small size of our task stimuli may naturally evoke local or near focus that would cause pupil constriction that could alternately be explained by accommodation rather than local attention. However, also observe smaller baseline pupil size during interstimulus periods for our local condition. Thus, there appears to be both cognitive or attentional influences on changes in pupil diameter beyond basic accommodation. The significant differences we observe in stimulus driven pupil response (e.g. constriction) also aligns with attentional processes such as the visual selection of local information as compared to global in hierarchical stimuli. Thus, there appears to be cognitive and attentional influences on changes in pupil diameter above and beyond accommodation. These alternative interpretations and potentially mixed findings when using pupillometry measurements underscores the potential impact of continued work in this domain. Additionally, regardless of whether accommodation or cognitive or perceptual influences should be the primary interpretation, Navon figures have been broadly used to capture perceptual biases and visual selection in both typical as well as atypical populations [1,2,4,53–55]. This study represents a novel application of Navon figures and pupillometry in the context of visual selection and demonstrates a significant difference in pupil response when selecting local versus global information in hierarchical stimuli. Findings presented here may help to interpret atypical pupil response during cognitive and perceptual tasks in clinical populations.

## Conclusion

The work presented here extends the literature on selective attention and represents a critical first step in the use of pupil response as an index of visuospatial selection and focused attention. Such findings may also be clinically relevant, particularly across disorders where atypical patterns of visual selection and differences in visual perceptual ability have been noted, such as within autism spectrum disorders (ASD) [56–63]. Other clinical populations face challenges sustaining attention (i.e. ADD/ADHD, schizophrenia, schizotypal personality) and have reported difficulties in narrowing their attention window to restrict and sustain spatial focus [64,65]. Atypical attentional functioning and deficits in maintaining attention in the context of global-local perception have also been reported in subcortical degenerative disorders such as Huntington’s and Parkinson’s disease [66–68]. Investigations of pupil response in the context of visual perception and global-local processing may point to perceptual and cognitive mechanisms underlying clinically-relevant cognitive and perceptual abilities. Finally, further research regarding the contribution of subcortical structures (i.e. the superior colliculus) on pupil response and in the early stages of visual perception may shed light on the underlying neural structures which guide perceptual biases, the ability to discern hierarchical or complex visual information, and lead to atypical patterns of visual attention [69,70]. Although data from clinical samples was not included in the current study, future iterations of this research would benefit from the inclusion of clinical populations in order to characterize atypical pupil response and the potential link aberrant perceptual skills.

## Supporting information

S1 Supporting information Supplementary eye blink analysis. A supplementary investigation of eye blinks that during baseline that would lead to a large amount of data loss and artificially reduce the estimation of pupil size during the baseline period. While eye blinks result in segments of missing data and have been shown to impede measures of pupil diameter as well as the interpolation of missing data, findings from this supplementary analysis confirm that eye blinks in the current study did not result in an artifact in reported pupil response data.
(DOCX)

S2 Supporting information Pupil response data. A .csv file containing task-induced pupil response data for both global and local conditions used in primary analysis that resulted in the reported results of a characteristic pupil constriction when identifying local information as compared to global information.
(CSV)
