# Supplementary material for: Task-induced pupil response and visual perception in adults
Source: PLoS One. 2018 Dec 26;13(12):e0209556. doi: 10.1371/journal.pone.0209556 (PMC6306195; doi:10.1371/journal.pone.0209556)
Supplement: S1 Supporting information — A supplementary investigation of eye blinks that during baseline that would lead to a large amount of data loss and artificially reduce the estimation of pupil size during the baseline period. While eye blinks result in segments of missing data and have been shown to impede measures of pupil diameter as well as the interpolation of missing data, findings from this supplementary analysis confirm that eye blinks in the current study did not result in an artifact in reported pupil response data. (DOCX) [file pone.0209556.s001.docx]

Supporting Information 1: Supplementary eye blink analysis

Given the relatively fast rise in pupil dilation during the first time bin, we also explored whether eye blinks were interrupting our pupil measurements during the baseline period and causing an artifact in our reported pupil response data, particularly within the initial dilation. That is, we wanted to determine whether blinks that occurred during baseline would lead to a large amount of data loss that artificially reduced the estimated pupil size during the baseline period. If this were the case, the fast rise in pupil dilation during the first time bin could be an artifact due to this data loss.

In pupillometry research, eye blinks are typically handled as segments of missing data since the pupil is occluded. Various interpolation or estimation methods can be implemented, as those described in the main text of the manuscript, to fill in these segments and approximate pupil response. Eye blinks also represent an early sensory response that may be especially sensitive to stimulus onset and offset as well as cognitive or perceptual processing. We extracted spontaneous eye blinks from the baseline period for all trials. Blinks were defined as continuous periods of missing eye gaze data of at least 100 msec and >500 msec. This definition of eye blinks was based upon previous eye tracking research and established infrared eye tracking norms [40].

We then counted the number of eye blinks during the baseline period that immediately preceded the onset of the stimulus across all trials. We found that no eye blinks occurred during the baseline period for the majority (>70%) of the trials across all participants. To reiterate in a slightly different way, a blink only occurred during the baseline period in less than 30% of all trials. The results of this analysis indicate that initial dilation in the first time bin is unlikely to be related to blinks immediately preceding the stimulus onset.

In addition to counting the total number of blinks across all trials for each participant, we also determined whether there were outlier subjects that could be driving an artifact. Outlier subjects were defined as individuals with an eye blink count ≥ 2 standard deviations from the group average across both conditions. Following identification of N=3 outlier subjects, we repeated our primary analyses with those individuals (n=3) excluded. These results are visualized and described in detail below and are identical to those already reported in the main manuscript. Thus, while eye blinks result in segments of missing data and have been shown to impede measures of pupil diameter as well as the interpolation of missing data, these findings confirm that eye blinks in the current study did not result in an artifact in reported pupil response data.


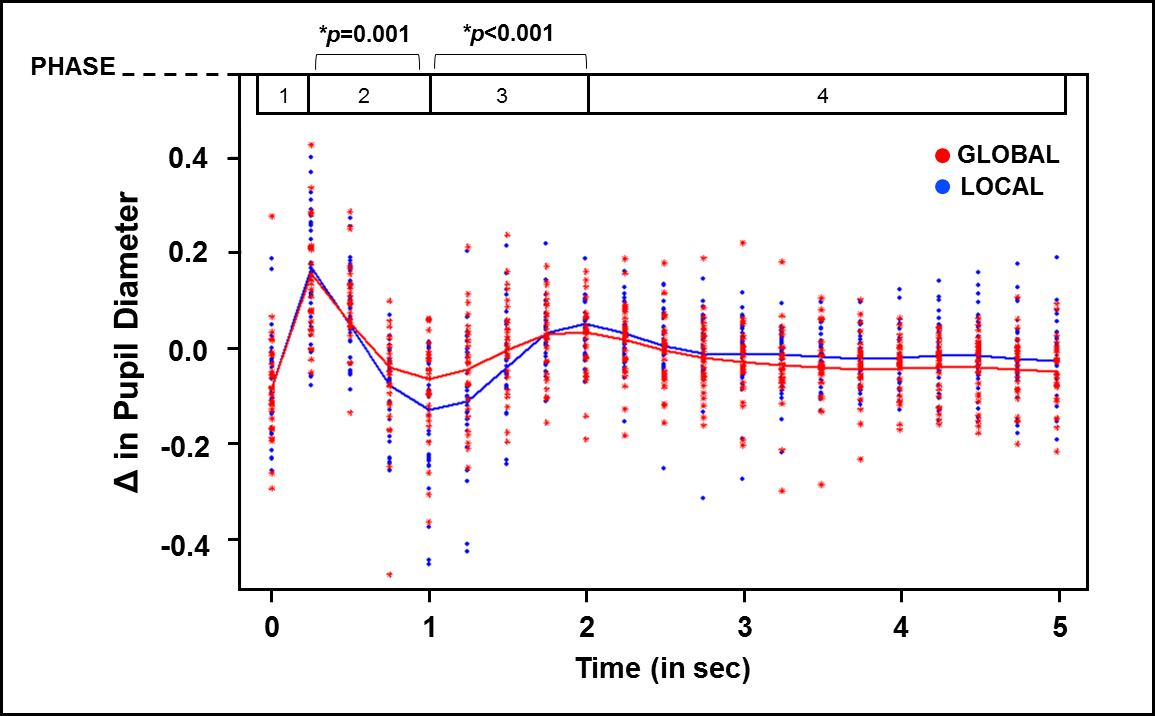


|  | Mean (st. dev.) | | | Paired t-test | | Bootstrap  (1000 resamples) |
| --- | --- | --- | --- | --- | --- | --- |
|  | Global Δ | Local Δ | Difference  (95% CI) | t(29) | *p*-value | *p*-value |
| Phase 1 | 0.22 (0.20) | 0.23 (0.23) | -0.01  (-0.07, 0.04) | -0.45 | 0.66 | 0.68 |
| Phase 2 | -0.22 (0.14) | -0.31 (0.20) | 0.09  (0.04, 0.14) | 3.5 | 0.002 | 0.001* |
| Phase 3 | 0.11 (0.11) | 0.19 (0.14) | -0.09  (-0.12, -0.05) | -5.2 | <0.001 | <0.001* |
| Phase 4 | -0.07 (0.12) | -0.06 (0.12) | -0.01  (-0.05, 0.03) | -0.45 | 0.65 | 0.68 |

In Phase 1, change in pupil dilation in the initial stage of the pupil response waveform did not differ between global and local conditions (Global Δ Value: 0.22 mm, Local Δ Value: 0.23 mm; t(29)= -0.45, *p*= 0.68, NS). In Phase 2, a rapid pupil constriction was observed in both conditions. This constriction was significantly larger for the local condition relative to the global condition (Global Δ Value: -0.22 mm, Local Δ Value: -0.31 mm; t(29)= 3.5, *p=*0.001). In Phase 3, pupil response dilated across both global and local conditions. Once again, significant differences in Phase 3 of the pupil response were present between global and local conditions (Global Δ Value: 0.11 mm, Local Δ Value: 0.19 mm; t(29)= -5.2, *p*<0.001), with a larger change in pupil diameter in the local condition relative to the global condition during this phase. In the final segment of the pupil response waveform (Phase 4), change in pupil size did not differ between conditions (Global Δ Value: -0.07 mm, Local Δ Value: -0.06 mm; t(29= -0.45, *p*= 0.68, NS).
